# Integrated analysis of the lncRNA-miRNA-mRNA network based on competing endogenous RNA in atrial fibrillation

**DOI:** 10.3389/fcvm.2023.1099124

**Published:** 2023-04-27

**Authors:** Manman Wang, Guoying An, Benxuan Wang, Yuanyuan Chen, Genli Liu, Xin Wang, Shuai Liu, Daozou Zhang, Dandan Sun, Yanyan Zhang, Tong Shen, Xiangting Li

**Affiliations:** ^1^ Jining Key Laboratory for Diagnosis and Treatment of Cardiovascular Diseases, Department of Cardiology, Affiliated Hospital of Jining Medical University, Jining, China; ^2^ Shandong Provincial Key Laboratory of Cardiac Disease Diagnosis and Treatment, Department of Cardiac Surgery, Affiliated Hospital of Jining Medical University, Jining, China; ^3^ Department of Neurology, Jinnan Hospital, Tianjin, China; ^4^ Admission and Patient Service Center, Affiliated Hospital of Jining Medical University, Jining, China

**Keywords:** atrial fibrillation, long non-coding RNAs, microRNAs, competing endogenous RNA, bioinformatics analysis

## Abstract

**Objective:**

Long non-coding RNAs (lncRNAs) play pivotal roles in the transcriptional regulation of atrial fibrillation (AF) by acting as competing endogenous RNAs (ceRNAs). In the present study, the expression levels of lncRNAs of sinus rhythm (SR) patients and AF patients were investigated with transcriptomics technology, and the lncRNA-miRNA-mRNA network based on the ceRNA theory in AF was elaborated.

**Methods:**

Left atrial appendage (LAA) tissues were obtained from patients with valvular heart disease during cardiac surgery, and they were divided into SR and AF groups. The expression characterizations of differentially expressed (DE) lncRNAs in the two groups were revealed by high-throughput sequencing methods. Gene Ontology (GO) and Kyoto Encyclopedia of Genes and Genomes (KEGG) pathway enrichment analyses were performed, and the lncRNA-miRNA-mRNA-mediated ceRNA network was constructed.

**Results:**

A total of differentially expressed 82 lncRNAs, 18 miRNAs, and 495 mRNAs in human atrial appendage tissues were targeted. Compared to SR patients, the following changes were found in AF patients: 32 upregulated and 50 downregulated lncRNAs; 7 upregulated and 11 downregulated miRNAs; and 408 upregulated and 87 downregulated mRNAs. A lncRNA-miRNA-mRNA network was constructed, which included 44 lncRNAs, 18 miRNAs, and 347 mRNAs. qRT-PCR was performed to verify these findings. GO and KEGG analyses suggested that inflammatory response, chemokine signaling pathway, and other biological processes play important roles in the pathogenesis of AF. Network analysis based on the ceRNA theory identified that lncRNA XR_001750763.2 and Toll-like receptor 2 (TLR2) compete for binding to miR-302b-3p. In AF patients, lncRNA XR_001750763.2 and TLR2 were upregulated, and miR-302b-3p was downregulated.

**Conclusion:**

We identified a lncRNA XR_001750763.2/miR-302b-3p/TLR2 network based on the ceRNA theory in AF. The present study shed light on the physiological functions of lncRNAs and provided information for exploring potential treatments for AF.

## 1. Introduction

Atrial fibrillation (AF) is a type of supraventricular arrhythmia characterized by rapid and disorderly atrial electrical activity, and it is one of the common arrhythmias found in the clinic. In recent years, the number of patients with AF has gradually increased, and a total of 59.7 million people worldwide have been reported to suffer from AF in 2019 (1). AF is a major disease that endangers human health, and it is a prominent cause of increased morbidity and mortality worldwide, especially as it initiates stroke, myocardial infarction, and heart failure, resulting in a significant global burden (2, 3). At present, antiarrhythmic drugs and ablation therapy have been effective strategies in the treatment of AF (4). However, the high recurrence rate of AF results in a poor prognosis and repeated hospitalization. The pathophysiological mechanism underlying AF is complex and remains unclear. Therefore, the exploration of specific new biomarkers and identification of the regulatory factors of early initiation and progression of AF remain a key focus in the prevention and treatment of AF.

Long noncoding RNAs (lncRNAs) are a class of natural nucleic acid molecules with lengths longer than 200 nucleotides, and they regulate gene expression at multiple levels. LncRNAs cannot encode proteins because they lack a conserved open reading frame (5). According to the different positions of adjacent protein-coding genes, lncRNAs can be divided into the following six categories: sense lncRNAs, antisense lncRNAs, intronic lncRNAs, bidirectional lncRNAs, intergenic lncRNAs, and enhancer lncRNAs (6). Many studies have confirmed that the expression of lncRNAs is cell- and tissue-specific, and their specific subcellular localization, highly conserved local sequence elements, and unique spatial secondary structures stimulate the interaction of lncRNAs with proteins, DNA, or RNA. Thus, lncRNAs play critical roles in the regulation of gene expression at the epigenetic, transcriptional, post-transcriptional modification, and translation levels (7–9).

The competitive endogenous RNA (ceRNA) hypothesis has been a research hotspot in recent years, in which lncRNAs competitively bind to microRNAs (miRNAs) through miRNA response elements (MREs), thereby inhibiting downstream target gene silencing by isolating miRNA from messenger RNA (mRNA) (10). Studies have found that lncRNAs act as ceRNAs participating in the occurrence and development of cardiovascular diseases. For example, lncRNA PVT1 has been shown to function as a ceRNA that competitively binds miR-128-3p to facilitate Sp1 expression, thereby promoting atrial fibrosis (11). Nevertheless, the ceRNA mechanisms associated with AF remain unclear, and the use of differentially expressed (DE) lncRNAs in left atrial appendage (LAA) tissue as diagnostic biomarkers for AF remains to be analyzed.

In the present study, we analyzed the lncRNA profiles in LAA tissues of patients with persistent AF by transcriptome sequencing, and we constructed an AF-related lncRNA-miRNA-mRNA network based on the ceRNA theory by bioinformatics methods to identify novel biomarkers with high sensitivity and specificity for the diagnosis of AF.

## 2. Materials and methods

### 2.1. Patients and sample collection

Patients undergoing cardiac surgery from January 2021 to April 2021 at the Affiliated Hospital of Jining Medical University (Shandong, China) were enrolled in the present study and divided into the AF group (*n* = 4) and sinus rhythm (SR) group (*n* = 4) based on electrocardiogram and past medical history. Patients with persistent AF were selected as the study population. The exclusion criteria for the patients were as follows: (1) previous history of coronary artery disease; (2) undergone radiofrequency (RF) catheter ablation; (3) left ventricular ejection fraction <40%; (4) severe infection or malignant disease, such as infective endocarditis; (5) hyperthyroidism; (6) stroke; and (7) severe liver damage and renal dysfunction. A small LAA tissue biopsy was collected for subsequent studies.

### 2.2. Histological and immunohistochemical analyses

One third of each LAA tissue was incubated in 4% paraformaldehyde for histopathological staining, and the remaining 2/3 atrial tissue was stored at −80°C for transcriptome sequencing and molecular detection verification. The LAA tissue was incubated in 4% paraformaldehyde for 24 h at room temperature, dehydrated, embedded in paraffin, and cut into 5 μm sections. After heating the sections at 60°C for 1 h, hematoxylin-eosin (H&E) staining, Masson's trichome staining, and immunohistochemical staining were performed. The sections were incubated with anti-collagen I (Servicebio, China; GB11022-3, 1:1,000 dilution) and anti-collagen III (Servicebio, China; GB111629, 1:500 dilution). All images (200x magnification) were acquired using a light microscope (Olympus, Tokyo, Japan), and the integral optical density (IOD) and the area of the granules were calculated. The mean optical density (MOD), defined as IOD/area, was used to compare the expression levels of the above proteins. Semiquantitative measurement was performed using Image-Pro Plus software (version 6.0, Media Cybernetics).

### 2.3. Transcriptome sequencing analyses

Eight samples (4 from each group) were sent to Shanghai OE Biomedical Science and Technology Company (Shanghai, China) for library construction by high-throughput sequencing technology. Total RNA was extracted using the TruSeq Stranded Total RNA Library Prep Kit, and the RNA integrity was assessed using an Agilent 2,100 Bioanalyzer (Agilent Technologies, Santa Clara, CA, USA). Samples with RNA Integrity Number (RIN) ≥ 7.0 were screened for subsequent analysis. The libraries were built using TruSeq Stranded Total RNA with Ribo-Zero Gold according to the manufacturer's instructions. Sequencing was performed on an Illumina HiSeq™ 2,500 sequencing platform, and 150 bp/125 bp paired-end reads were generated.

### 2.4. LncRNA prediction and differential expression analyses

To screen out the candidate lncRNA transcripts, merged transcripts were compared with the gene annotation information of the reference sequences by Cuffcompare software. Transcripts with lengths >200 bp and exon number ≥2 were selected for subsequent screening. Fragments per kilobase million (FPKM) (12) was used to calculate the transcript expression. The estimateSizeFactors function of the DESeq package in R was used to normalize the counts, and the nbinomTest function was used to calculate the *p* value and fold change (FC) values for the comparison. Screening criteria of differential transcripts were set as *p* ≤ 0.05 and |log2FC| > 1.

### 2.5. CeRNA network construction

**Figure 1:**
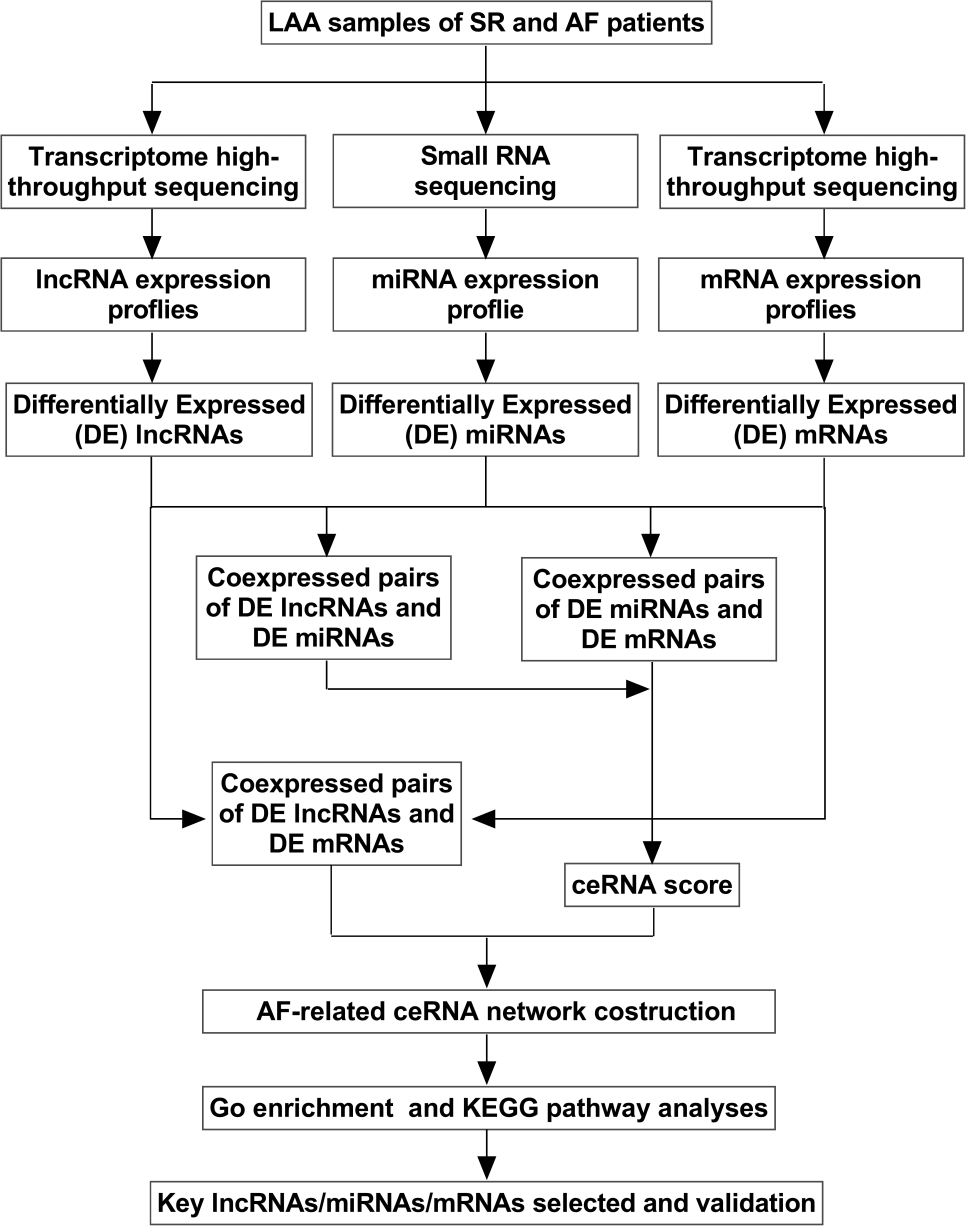
Flow chart of biological information analysis. LAA, left atrial appendage tissue; SR, sinus rhythm; AF, atrial fibrillation; GO, Gene Ontology; KEGG, Kyoto Encyclopedia of Genes and Genomes.

To construct the lncRNA-miRNA-mRNA network, we performed miRNA sequencing and mRNA sequencing. The coexpression relationships of differentially expressed (DE) miRNA-lncRNA were screened to predict their regulatory correlation according to specific steps. Pearson's r was used to calculate the correlation between DEmiRNAs and DElncRNAs, and the negative regulatory relationship pairs were then screened. The interactions between the miRNAs and lncRNAs were predicted using the miRanda (http://www.microrna.org/microrna/getMirnaForm.do). Similarly, miRNA-mRNA relationship pairs were obtained, and ceRNA scores were calculated between the two ceRNAs (mRNAs and lncRNAs) according to the MuTaME method. Based on the ceRNA hypothesis, lncRNAs act as endogenous miRNA sponges to regulate the expression and degradation of targeted mRNAs, and the expression levels of lncRNAs and mRNAs should be positively correlated (13). Therefore, positive mRNA-lncRNA coexpression relationship pairs were obtained. Finally, the ceRNA relationship pairs with high reliability were obtained, and the initial ceRNA network was built using Cytoscape software (http://cytoscape.org/). The applied flow chart is shown in Figure 1.

### 2.6. Function analyses of differentially expressed genes (DEGs) in the ceRNA network

To elucidate the biological function and features of DEGs in the ceRNA network, Gene Ontology (GO; http://geneontology.org/) (14) and Kyoto Encyclopedia of Genes and Genomes (KEGG; https://www.kegg.jp/) (15) analyses were performed using the hypergeometric distribution test, and *p* < 0.05 was considered significantly enriched.

### 2.7. Validation of DElncRNA, DEmiRNA, and DEmRNA expression

Total RNA was extracted from LAA tissues using TRIzol® reagent (Invitrogen), and the extracted RNA was reverse transcribed into cDNA using a FastQuant RT Kit (TianGen, Beijing, China). qRT-PCR was performed to detect the relative expression levels of lncRNAs, miRNAs, and mRNAs using a SYBR Green PCR kit (TransGen, Beijing, China) according to the manufacturer's instructions. PCR amplification was performed using the following thermocycler program: denaturation at 95°C for 10 min; and 40 cycles of 95°C for 30 s, 58–60°C for 30 s, and 72°C for 30 s The PCR assays were performed using a CFX Connect Real-Time System (Bio-Rad, USA). Glyceraldehyde 3-phosphate dehydrogenase (GAPDH) was used as an internal reference for mRNA expression, and U6 snRNA was used as an internal reference for lncRNA and miRNA expression. The primer sequences are listed in Table 1. The relative quantitative expression levels of the genes were analyzed using the 2^−*ΔΔ*Ct^ method. All experiments were performed in triplicate.

**Table 1:** Primers used in the present study.

Primer	species	Sequence (5’→3’)	Tm (°C)
XR_001750763.2	Human	F: ACCGGCGCATATTCAAAACG	60.0
		R: TTAGCCCTGCTTTTTGCTCTCT	
XR_932105.2	Human	F: CGTTCCCCTTGTTATTGCCAG	58.0
		R: CTGTTTGTAAGGTTTGGGGGAACT	
NR_038446.1	Human	F: AGCTTTGCTCACTCATCCCTTC	60.0
		R: GGAGTGGGGCAACAGCATA	
ENST00000447009	Human	F: CCCTTCCATTTTTCCTGGGGTG	60.0
		R: GAGCATTCCCGAAATCCAGCTT	
Has-miR-512-3p	Human	AAGUGCUGUCAUAGCUGAGGUC	60.0
Has-miR-302c-3p	Human	UAAGUGCUUCCAUGUUUCAGUGG	60.0
Has-miR-302b-3p	Human	UAAGUGCUUCCAUGUUUUAGUAG	60.0
U6	Human	F: CAGCACATATACTAAAATTGGAACG	60.0
		R: ACGAATTTGCGTGTCATCC	
TLR2	Human	F: TGCATTCCCAAGACACTGGA	60.0
		R: GGGAGGCATCTGGTAGAGTC	
JAK3	Human	F: CTCTATGCCTGCCAAGACCC	60.0
		R: GGCACCTGTATTGTCGCCTA	
CCL5	Human	F: CGAAAGAACCGCCAAGTGTG	60.0
		R: CGGGTGGGGTAGGATAGTGA	
VCAM1	Human	F: TGCAAGTCTACATATCACCCAAGAA	60.0
		R: GTAGACCCTCGCTGGAACAG	
GAPDH	Human	F: GCACCGTCAAGGCTGAGAAC	60.0
		R: TGGTGAAGACGCCAGTGGA	

### 2.8. Statistical analysis

Statistical analysis was performed using SPSS 22.0 (SPSS Inc., Chicago, IL, USA), and GraphPad Prism 8.0 (GraphPad, San Diego, CA, USA) was used to generate the plots. The continuous variables are expressed as the mean ± standard error of the mean (SEM), and the differences between the two groups were statistically analyzed using a two-tailed Student's t-test. The categorical variables are presented as the number and percentage, and they were analyzed by Fisher's exact test. *p* < 0.05 was considered statistically significant.

## 3. Results

### 3.1. General clinical characteristics of the study subjects

The general clinical data for the SR and AF patients are shown in Table 2. There were no differences in age, gender, body mass index (BMI), blood pressure (BP), renal function, liver function, blood lipids, hypertension, diabetes mellitus, stroke, right atrial diameter (RAD), or left ventricular end-diastolic dimension (LVDd) between the two groups. However, the AF group had a higher left atrial diameter (LAD) (*p* < 0.05) and lower left ventricular ejection fraction (LVEF) (*p* < 0.05) compared to the SR group, which suggested that AF may lead to decreased cardiac function.

**Table 2:** Comparison of clinical characteristics between the two groups.

Characteristics	SR patients (*n* = 4)	AF patients (*n* = 4)	*p*-value
Age (years)	60.75 ± 4.49	60 ± 7.71	0.889
Male, *n* (%)	3 (75.0)	1 (25.0)	0.486
BMI (kg/m^2^)	22.03 ± 1.47	26.31 ± 5.71	0.255
Congestive heart failure	1 (25.0)	1 (25.0)	1.000
Hypertension, *n* (%)	0 (0)	0 (0)	N/A
Diabetes mellitus, *n* (%)	0 (0)	1 (25.0)	1.000
Stroke, *n* (%)	0 (0)	0 (0)	N/A
HR (bpm)	80.00 ± 11.38	90.75 ± 23.89	0.508
SBP (mmHg)	138.25 ± 11.26	124.25 ± 12.03	0.191
DBP (mmHg)	68.00 ± 9.06	76.50 ± 15.98	0.453
Cr (umol/l)	63.18 ± 14.03	58.35 ± 9.33	0.638
ALT(U/l)	14.78 ± 4.19	27.55 ± 11.72	0.126
AST(U/l)	18.00 ± 3.39	25.25 ± 7.05	0.156
TC (mmol/l)	4.16 ± 0.45	3.96 ± 1.30	0.812
TG (mmol/l)	0.94 ± 0.21	0.97 ± 0.15	0.845
HDL-C (mmol/l)	1.19 ± 0.06	1.13 ± 0.24	0.690
LDL-C (mmol/l)	2.70 ± 0.34	2.54 ± 1.00	0.808
RAD (mm)	34.00 ± 2.92	45.50 ± 7.63	0.051
LAD (mm)	42.25 ± 8.10	64.25 ± 12.70	0.045*
LVDd (mm)	52.00 ± 12.06	46.50 ± 4.33	0.485
LVEF (%)	61.25 ± 1.30	54.25 ± 2.38	0.004*

SR, sinus rhythm; AF, atrial fibrillation; BMI, body mass index; HR, heart rate; SBP, systolic blood pressure; DBP, diastolic blood pressure; Cr, creatinine; ALT, alanine aminotransferase; AST, aspartate transaminase; TC, total cholesterol; TG, total glyceride; HDL-C, high-density lipoprotein cholesterol; LDL-C, low-density lipoprotein cholesterol; RAD, right atrial diameter; LAD, left atrial diameter; LVDd, left ventricular end-diastolic dimension; LVEF, left ventricular ejection fractions. N/A, not applicable. Values are presented as means ± SEM or number (%).

* *p *< 0.05.

### 3.2. Fibrotic changes occur in LAA tissues

**Figure 2:**
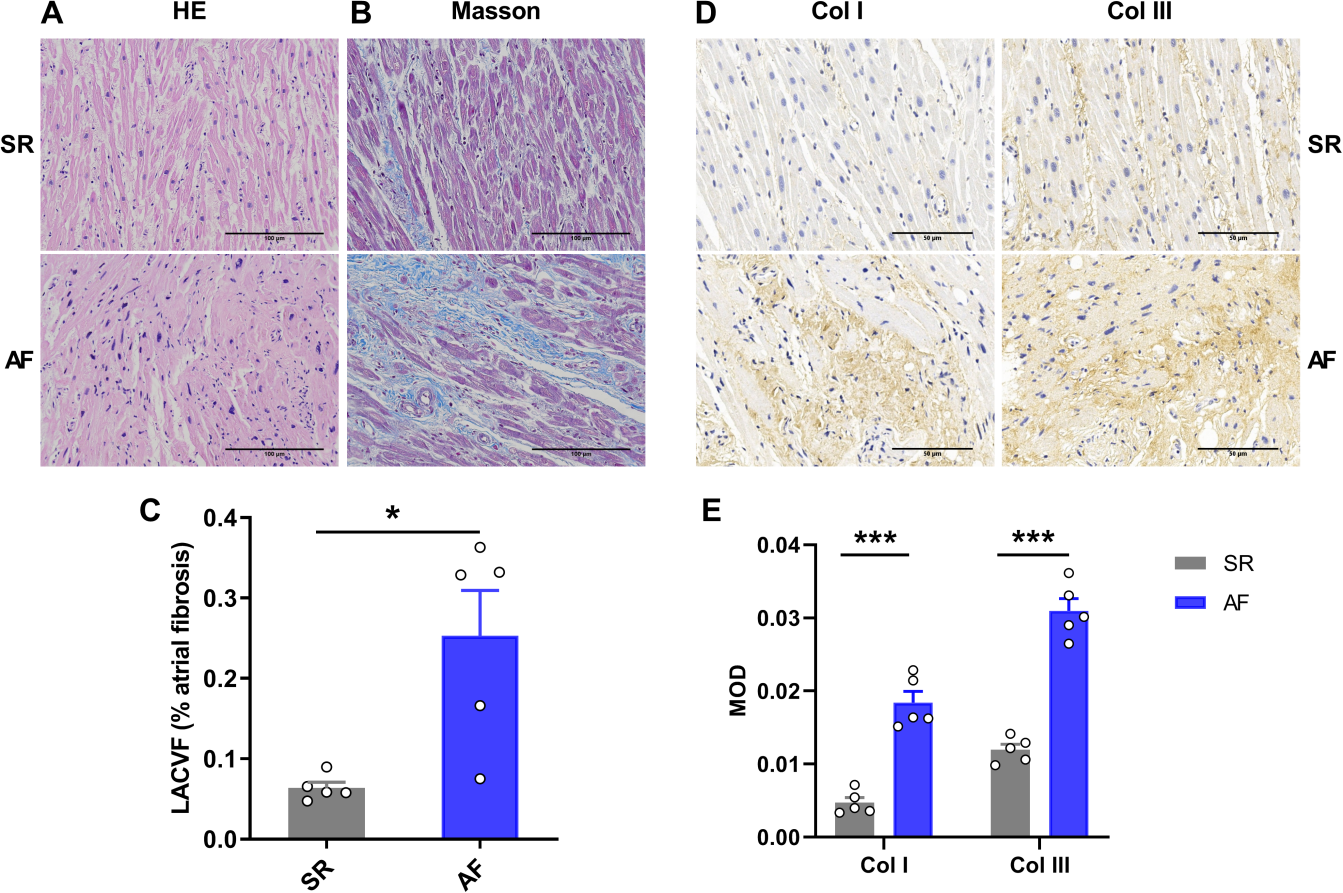
Atrial fibrosis occurs in patients with atrial fibrillation. (**A**) HE staining of LAA tissue in the SR and AF groups. (**B**) Masson's Trichrome staining in the two groups. (**C**) Comparison of left atrial collagen volume fraction between the SR and AF groups. (**D,E**) Immunohistochemistry staining and comparison of collagen I and III between the SR and AF groups. SR, sinus rhythm. AF, atrial fibrillation. Col I, collagen I; Col III, collagen III. *p < 0.05 and ***p < 0.001 compared to the SR group.

H&E staining showed that atrial myocytes in the SR group were arranged in an orderly manner with normal shape and size. Compared to the SR group, the myocardium in AF patients was ruptured, and the morphology of the atrial myocytes was abnormal, mainly manifested as a disordered arrangement of atrial myocytes and variable nuclear size (Figure 2A). Masson's trichrome staining also showed that more collagen was deposited in the intercellular spaces of atrial myocytes in the AF group compared to the SR group (Figure 2B). In addition, the left atrial collagen volume fraction (LACVF) in the AF group was significantly higher than that in the SR group (SR vs. AF: 6.39 ± 1.42% vs. 25.31 ± 11.26%, *p* < 0.05) (Figure 2C), which indicated that the atrial fibrous tissues in the AF group were significantly increased. Moreover, immunohistochemistry (IHC) analysis indicated that the two major proteins in the extracellular matrix, collagen type I and III, were significantly upregulated in the atrial tissues of AF patients compared to the SR group (Figures 2D,E). These data indicated that the atrial tissues of patients with AF have obvious fibrotic changes.

### 3.3. Characterization of human lncRNAs

**Figure 3:**
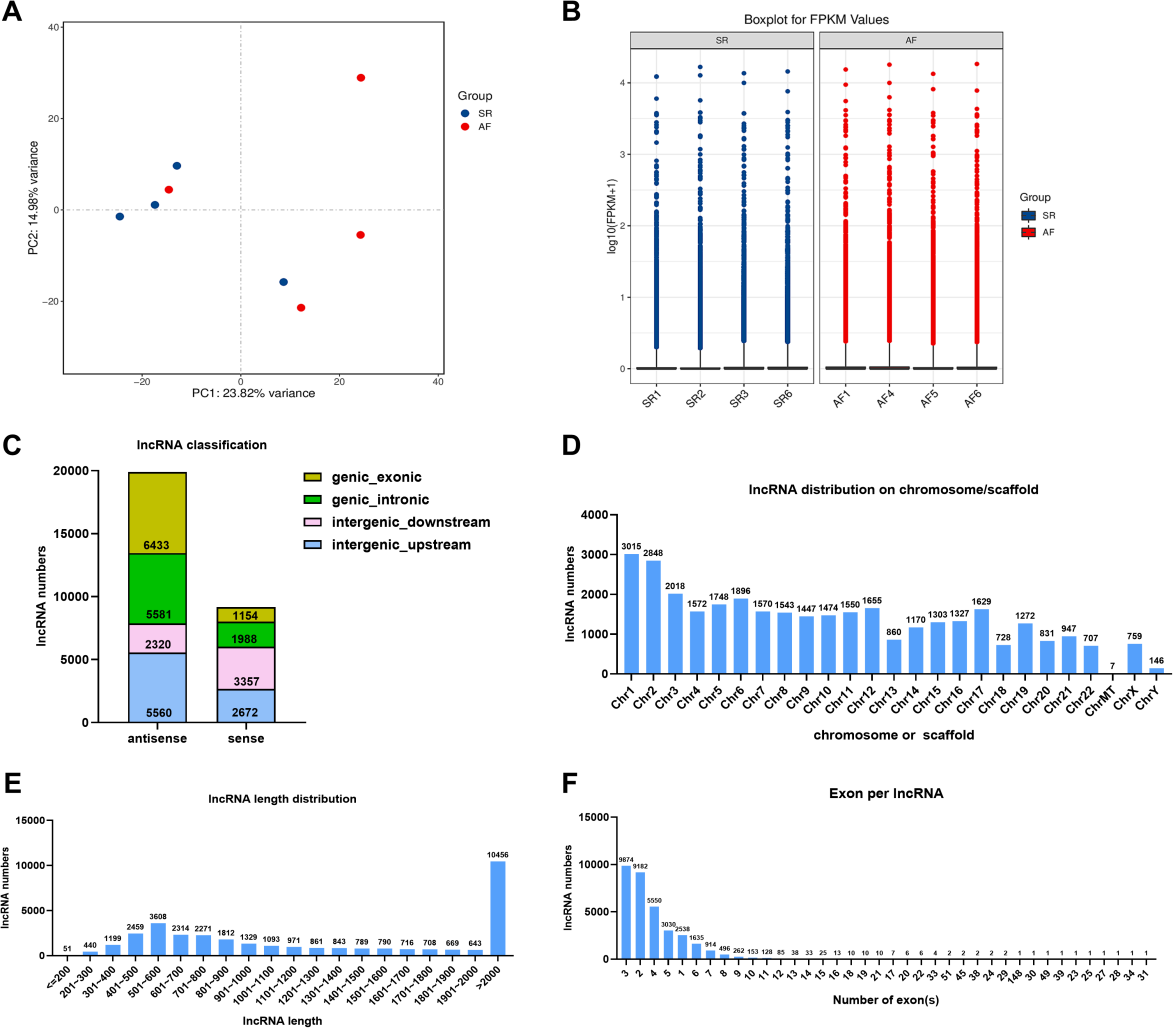
Transcriptome sequencing analysis of DE lncRNAs in the SR and AF groups. (**A**) Principal component analysis (PCA) of SR and AF patient samples. (**B**) Boxplot for FPKM values of lncRNAs in each sample. (**C**) Percentage of different types of lncRNAs. (**D**) Chromosomal distribution of the lncRNAs. (**E**) LncRNA lengths in the two groups. (**F**) Statistical diagram of lncRNA exon numbers.

In the present study, whole transcriptome sequencing of eight samples obtained a total of 109.46 G clean data, among which the effective data volume of each sample was distributed between 12.56 and 14.41 G. Q30 bases ranged from 95.32% to 95.57% with an average GC content of 48.53%. The contrast rates to the reference genome of these samples were between 97.90% to 98.20%. Principal component analysis (PCA) of the SR and AF samples indicated that each group was clustered (Figure 3A). A total of 34,022 lncRNAs were detected, box-whisker plot was used to shown the lncRNA expression distribution in each sample in terms of log10(FPKM + 1) (Figure 3B). To understand the genomic characteristics of the lncRNAs expressed in AF, we examined their classifications, distribution of their length, chromosome distribution, and their exon numbers. According to their direction, type, and location relative to known protein-coding transcripts, the lncRNAs were classified into several types with 19,894 (68.45%) antisense lncRNAs and only 9,171 (31.55%) sense lncRNAs (Figure 3C). These lncRNAs were widely distributed in all chromosomes (Figure 3D), and most lncRNAs (69.12%) were 200 to 2,000 base pairs (bp) in length (Figure 3E). Approximately 7.46% of lncRNAs were composed of a single exon, and the remaining lncRNAs had multiple exons (Figure 3F). Based on this, we speculate that lncRNAs are highly enriched in AF tissues and have the potential to participate in the biological function of AF.

### 3.4. Expression profiles of lncRNAs, miRNAs, and mRNAs

**Figure 4:**
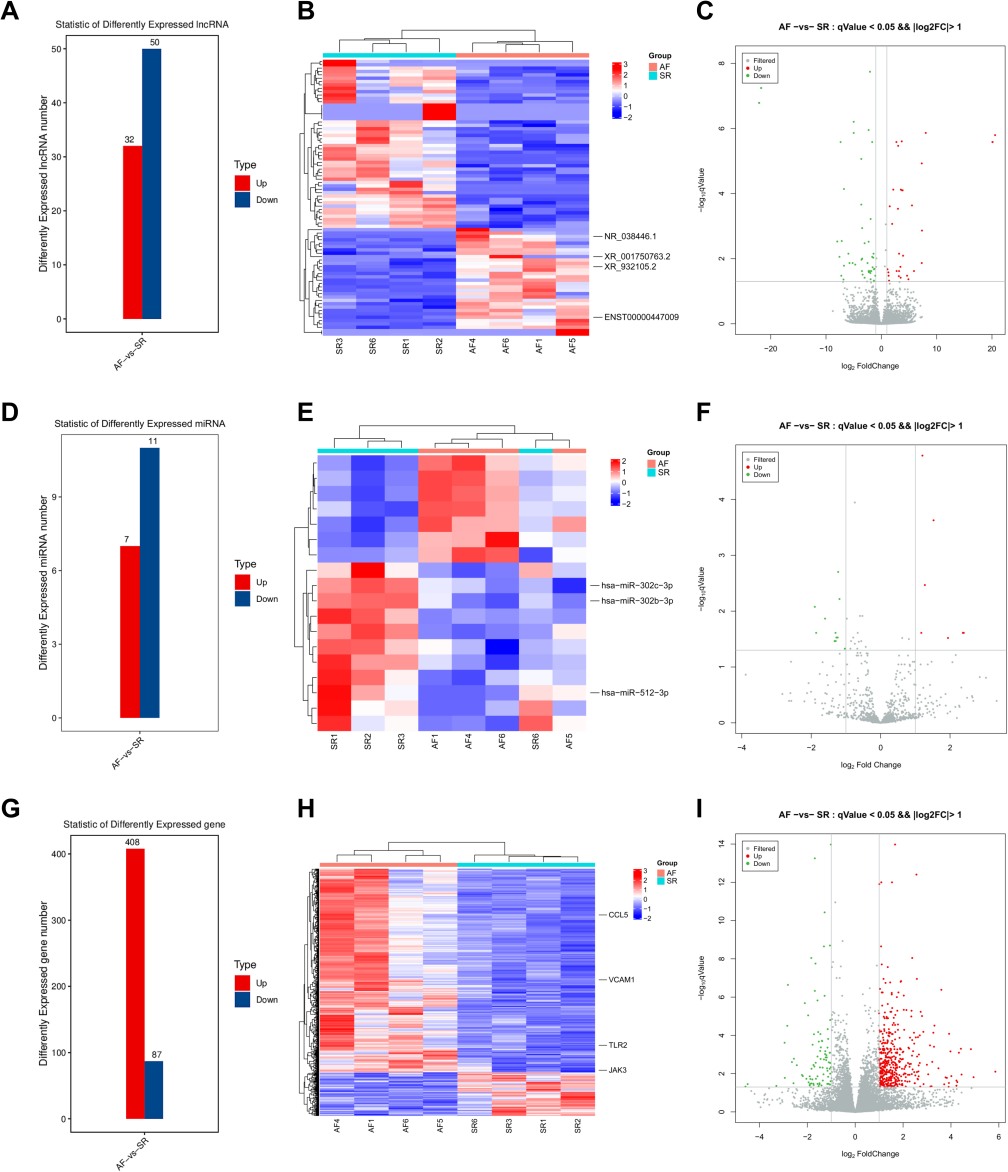
DElncRNAs, DEmiRNAs, and DEmRNAs. The DElncRNA number (**A**), heatmap (**B**), and volcano plot (**C**) comparisons between the two groups. The DEmiRNA number (**D**), heatmap (**E**), and volcano plot (**F**) comparisons between the two groups. The DEmRNA number (**G**), heatmap (**H**), and volcano plot (**I**) comparisons between the two groups.

In the present study, a total of 82 lncRNAs, 18 miRNAs, and 495 mRNAs were significantly DE with |log2FC| > 1 (*p* < 0.05) in LAA samples of AF patients compared to SR patients (Supplementary Tables S1–S3). Heatmaps and volcano maps were generated to visualize the overall distribution of DElncRNAs, DEmiRNAs, and DEmRNAs (Figure 4). Among the 82 lncRNAs, 32 lncRNAs were upregulated, and 50 lncRNAs were downregulated (Figures 4A–C). Among the 18 miRNAs, 7 miRNAs were upregulated, and 11 miRNAs were downregulated (Figures 4D–F). Among the 495 mRNAs, 408 mRNAs were upregulated, and 87 mRNAs were downregulated (Figures 4G–I).

### 3.5. Construction of the ceRNA network

**Figure 5:**
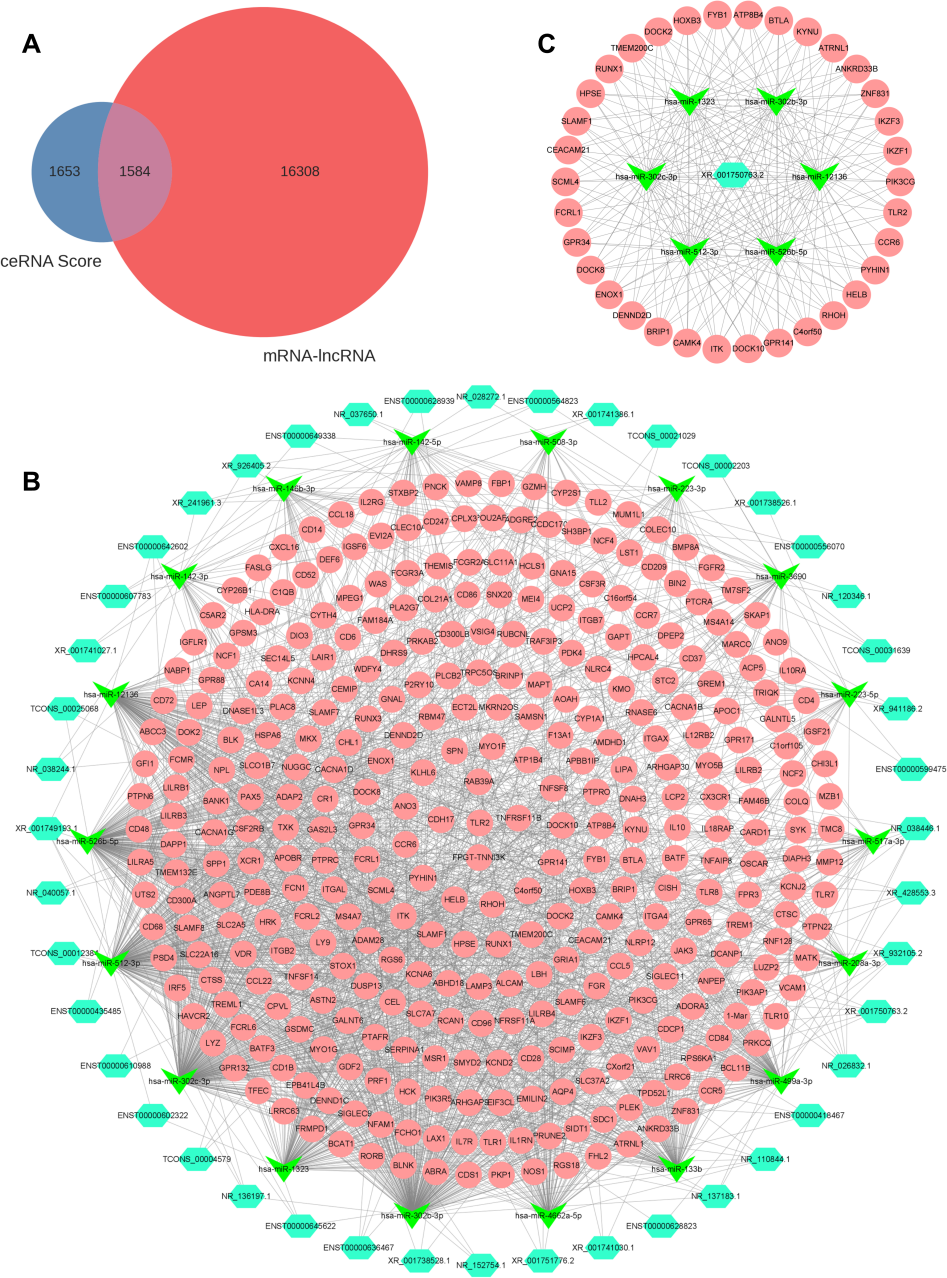
LncRNA-miRNA-mRNA regulatory network. (**A**) Venn diagram showing results of the ceRNA score and coexpression analysis. (**B**) Total lncRNA-miRNA-mRNA regulatory network. Light blue, green, and pink represent lncRNAs, miRNAs, and mRNAs, respectively. (**C**) Top 200 DElncRNA-DEmiRNA-DElncRNA axes in the ceRNA network. DE, differentially expressed.

The intersection between the ceRNA score and the DEmiRNA-DElncRNA coexpression results identified a total of 1,584 DElncRNA-DEmiRNA-DEmRNA relationship pairs after filtering (Figure 5A and Supplementary Tables S4, S5). A total of 44 lncRNAs, 18 miRNAs, and 347 mRNAs were involved in the lncRNA-miRNA-mRNA network (Figure 5B and Supplementary Table S6), and the top 200 DElncRNA-DEmiRNA-DElncRNA axes with 1 DElncRNA, 6 DEmiRNAs, and 34 DEmRNAs were identified using Cytoscape software (Figure 5C).

### 3.6. Functional enrichment analyses

**Figure 6:**
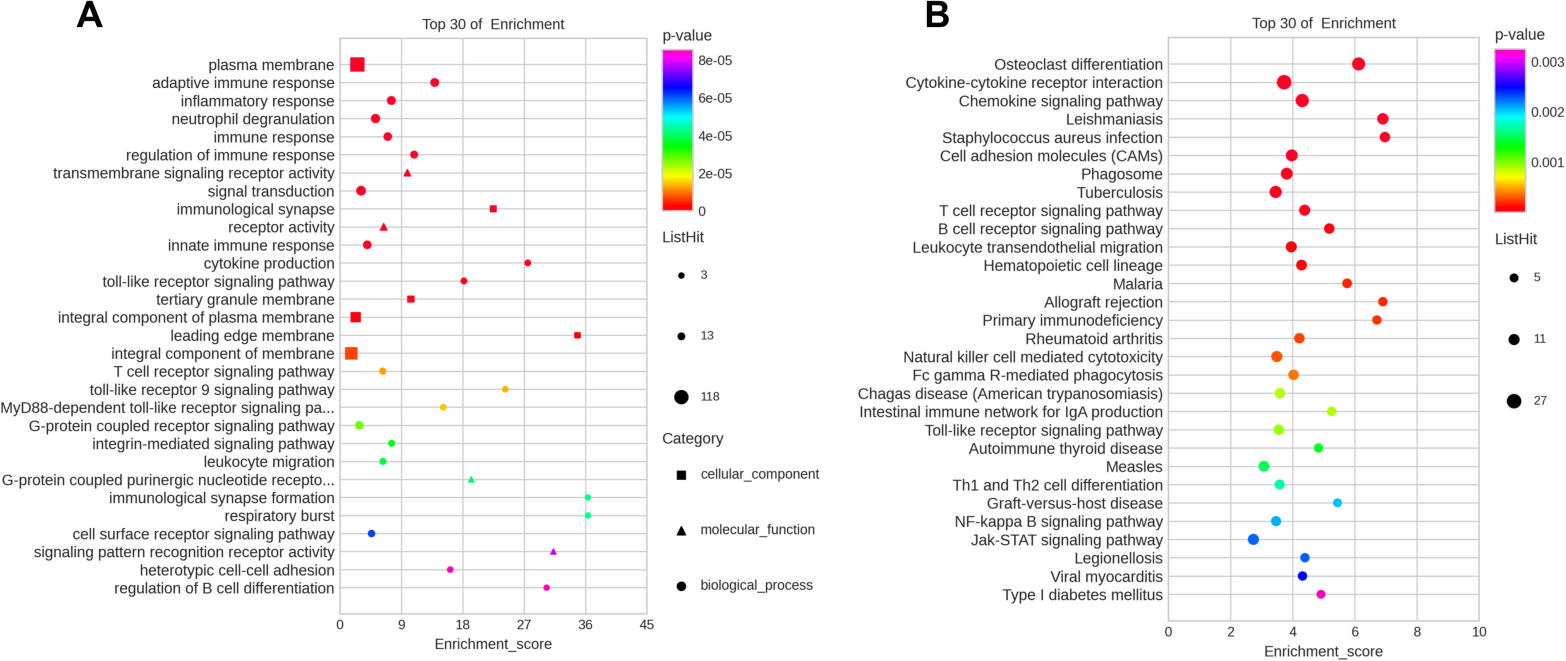
Top 30 most enriched GO and KEGG terms by coexpression target mRNAs. (**A**) Top 30 enriched terms according to GO analysis. (**B**) Top 30 signaling pathways according to KEGG analysis.

To further investigate the biological functions of the DEGs in the ceRNA network, GO and KEGG enrichment analyses were performed (Supplementary Tables S7, S8). GO enrichment analysis revealed that the DEGs were enriched in three GO classifications, namely, biological process, cellular component, and molecular function. The top 30 terms with the smallest *p* value in the GO enrichment analysis were visualized using a bubble plot, and the top three terms were plasma membrane, adaptive immune response, and inflammatory response (Figure 6A and Table 3). A bubble plot was also used to visualize the top 30 KEGG pathways, and the top three pathways with the lowest *p* value were osteoclast differentiation, cytokine receptor interaction, and chemokine signaling pathway (Figure 6B and Table 3). In addition, the Toll-like receptor signaling pathway and nuclear factor kappa B (NF-κB) signaling pathway were also suggested to be involved in the progression of AF, which was consistent with previous studies (16).

**Table 3:** Top 30 enriched GO terms and KEGG pathways of DEGs in the ceRNET.

Term_ID	Term_description	Genes number	*p*-value	FDR
GO:0005886	plasma membrane	118	5.58 × 10^−25^	1.03 × 10^−22^
GO:0002250	adaptive immune response	22	1.93 × 10^−19^	1.44 × 10^−16^
GO:0006954	inflammatory response	23	3.96 × 10^−14^	1.48 × 10^−11^
GO:0043312	neutrophil degranulation	27	1.88 × 10^−12^	4.67 × 10^−10^
GO:0006955	immune response	20	7.74 × 10^−12^	1.44 × 10^−9^
GO:0050776	regulation of immune response	14	2.96 × 10^−11^	4.41 × 10^−9^
GO:0004888	transmembrane signaling receptor activity	12	2.52 × 10^−9^	6.34 × 10^−7^
GO:0007165	signal transduction	32	1.20 × 10^−8^	1.49 × 10^−6^
GO:0001772	immunological synapse	7	1.26 × 10^−8^	1.17 × 10^−6^
GO:0004872	receptor activity	13	1.15 × 10^−7^	1.45 × 10^−5^
GO:0045087	innate immune response	20	1.43 × 10^−7^	1.52 × 10^−5^
GO:0001816	cytokine production	5	4.98 × 10^−7^	4.65 × 10^−5^
GO:0002224	toll-like receptor signaling pathway	6	6.03 × 10^−7^	4.99 × 10^−5^
GO:0070821	tertiary granule membrane	8	8.54 × 10^−7^	5.26 × 10^−5^
GO:0005887	integral component of plasma membrane	38	1.57 × 10^−6^	7.28 × 10^−5^
GO:0031256	leading edge membrane	4	2.39 × 10^−6^	8.84 × 10^−5^
GO:0016021	integral component of membrane	74	6.94 × 10^−6^	0.000214035
GO:0050852	T cell receptor signaling pathway	9	1.30 × 10^−5^	0.000920703
GO:0034162	toll-like receptor 9 signaling pathway	4	1.40 × 10^−5^	0.000920703
GO:0002755	MyD88-dependent toll-like receptor signaling pathway	5	1.48 × 10^−5^	0.000920703
GO:0007186	G-protein coupled receptor signaling pathway	20	2.71 × 10^−5^	0.00155363
GO:0007229	integrin-mediated signaling pathway	7	3.53 × 10^−5^	0.001883319
GO:0050900	leukocyte migration	8	3.83 × 10^−5^	0.001898271
GO:0045028	G-protein coupled purinergic nucleotide receptor activity	4	4.04 × 10^−5^	0.003393147
GO:0045730	respiratory burst	3	4.33 × 10^−5^	0.001898271
GO:0001771	immunological synapse formation	3	4.33 × 10^−5^	0.001898271
GO:0007166	cell surface receptor signaling pathway	10	6.18 × 10^−5^	0.002561792
GO:0008329	signaling pattern recognition receptor activity	3	7.76 × 10^−5^	0.004887451
GO:0034113	heterotypic cell-cell adhesion	4	8.54 × 10^−5^	0.003035922
GO:0045577	regulation of B cell differentiation	3	8.55 × 10^−5^	0.003035922
KEGG pathway				
path:hsa04380	Osteoclast differentiation	19	1.71 × 10^−10^	3.61 × 10^−8^
path:hsa04060	Cytokine-cytokine receptor interaction	27	2.39 × 10^−9^	2.53 × 10^−7^
path:hsa04062	Chemokine signaling pathway	20	2.99 × 10^−8^	2.11 × 10^−6^
path:hsa05140	Leishmaniasis	12	1.16 × 10^−7^	6.12 × 10^−6^
path:hsa05150	Staphylococcus aureus infection	9	4.32 × 10^−6^	0.000182393
path:hsa04514	Cell adhesion molecules (CAMs)	14	1.03 × 10^−5^	0.000361002
path:hsa04145	Phagosome	14	1.65 × 10^−5^	0.00049856
path:hsa05152	Tuberculosis	15	2.68 × 10^−5^	0.000706923
path:hsa04660	T cell receptor signaling pathway	11	3.73 × 10^−5^	0.000874414
path:hsa04662	B cell receptor signaling pathway	9	5.20 × 10^−5^	0.001097399
path:hsa04670	Leukocyte transendothelial migration	11	9.72 × 10^−5^	0.001820171
path:hsa04640	Hematopoietic cell lineage	10	0.000103517	0.001820171
path:hsa05144	Malaria	7	0.000184759	0.002903922
path:hsa05330	Allograft rejection	6	0.000192677	0.002903922
path:hsa05340	Primary immunodeficiency	6	0.000226498	0.003186066
path:hsa05323	Rheumatoid arthritis	9	0.000261516	0.003448736
path:hsa04650	Natural killer cell mediated cytotoxicity	11	0.000298299	0.003702418
path:hsa04666	Fc gamma R-mediated phagocytosis	9	0.000368875	0.004324039
path:hsa05142	Chagas disease (American trypanosomiasis)	9	0.000863427	0.009315152
path:hsa04672	Intestinal immune network for IgA production	6	0.000886799	0.009315152
path:hsa04620	Toll-like receptor signaling pathway	9	0.0009271	0.009315152
path:hsa05320	Autoimmune thyroid disease	6	0.001385996	0.01329296
path:hsa05162	Measles	10	0.001497073	0.013734018
path:hsa04658	Th1 and Th2 cell differentiation	8	0.001694964	0.014901556
path:hsa05332	Graft-vs.-host disease	5	0.00204633	0.016968598
path:hsa04064	NF-kappa B signaling pathway	8	0.002090917	0.016968598
path:hsa04630	Jak-STAT signaling pathway	11	0.002260369	0.017200992
path:hsa05134	Legionellosis	6	0.002282596	0.017200992
path:hsa05416	Viral myocarditis	6	0.002504793	0.018224532
path:hsa04940	Type I diabetes mellitus	5	0.003247711	0.022725173

FDR, false discovery rate. DEGs, differentially expressed genes; ceRNET, ceRNA network.

### 3.7. Validation of RNA expression in the ceRNA network

**Figure 7:**
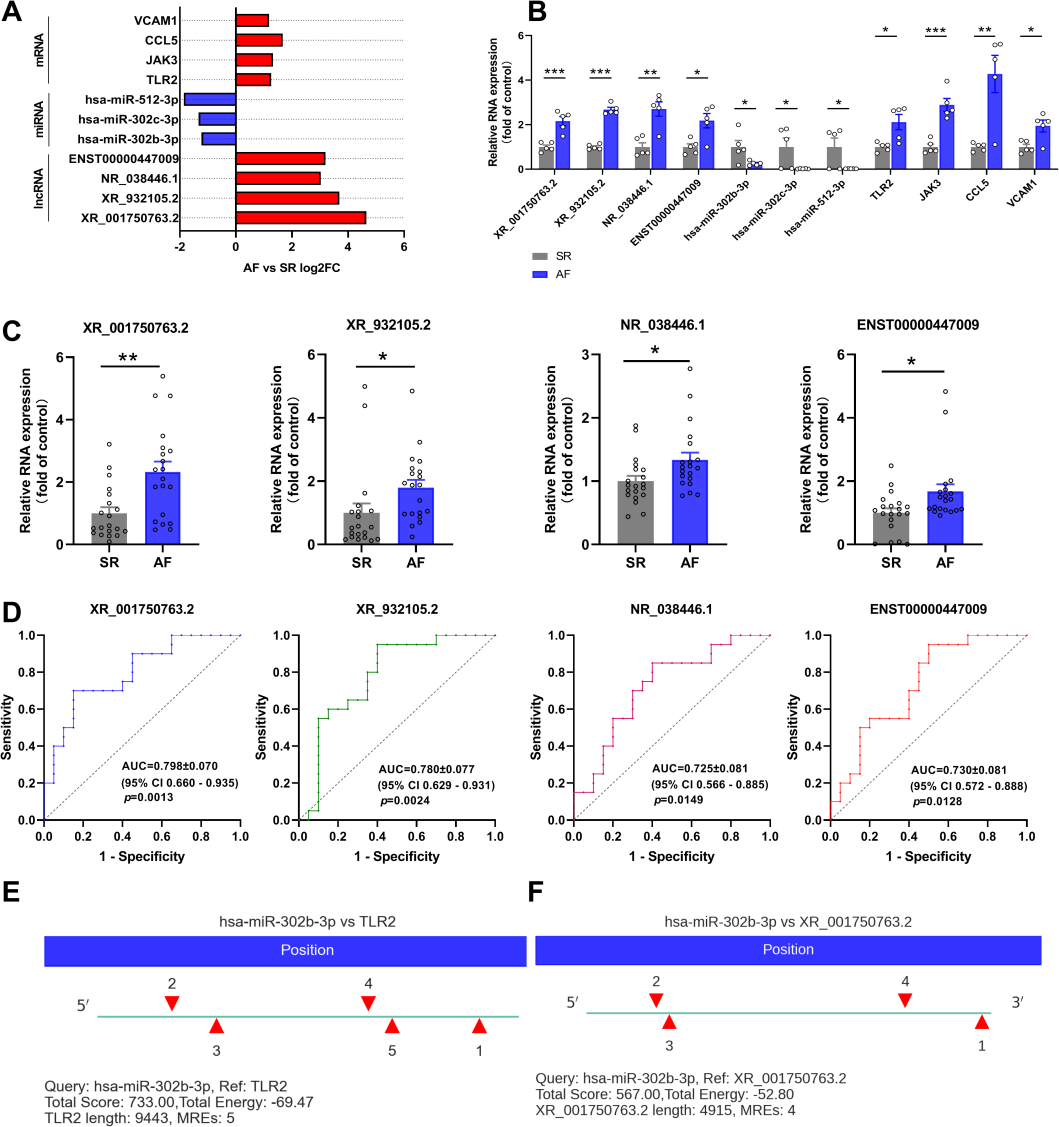
Verification of the differentially expressed (DE) lncRNAs, miRNAs, and mRNAs. (**A**) Fold change for each lncRNA, miRNA, and mRNA as indicated by RNA sequencing. (**B**) qRT-PCR was used to verify the selected DE lncRNAs, miRNAs and mRNAs in left atrial appendage (LAA) tissues. (**C**) qRT-PCR was used to verify the selected DE lncRNAs in the peripheral blood of patients. (**D**) Receiver operating characteristic curve analysis of each lncRNA to distinguish patients with AF from the SR. AUC, area under the curve; CI, confidence interval. (**E,F**) The miR-302b-3p binding sites on XR_001750763.2 and TLR2 were predicted by miRanda.

Based on the ceRNA theory, the expression trend of screened lncRNAs and mRNAs was similar, whereas the expression trend of miRNAs was the opposite. To detect the accuracy of sequencing results, the sequences of the top two upregulated lncRNAs (XR_001750763.2 and XR_932105.2) with the highest ceRNA score and other randomly selected RNAs, including two lncRNAs (NR_038446.1 and ENST00000447009), three miRNAs (hsa-miR-302b-3p, hsa-miR-302c-3p, and hsa-miR-512-3p), and four mRNAs (Toll-like receptor 2, TLR2; Janus kinase 3, JAK3; C-C motif chemokine ligand 5, CCL5; and vascular cell adhesion molecule 1, VCAM1) in the ceRNA network were validated by qRT-PCR (Figure 7B). The results were consistent with the high-throughput sequencing analysis (Figure 7A and Table 4). To further verify the diagnostic value of the four selected lncRNAs, the peripheral blood of patients (20 SR patients and 20 AF patients) was collected, the inclusion and exclusion criteria are the same as step 2.1, and RNA was extracted for qRT-PCR detection. The general clinical data of these patients are shown in Table 5. The expression levels of XR_001750763.2, XR_932105.2, NR_038446.1, and ENST00000447009 were significantly upregulated in AF patients (Figure 7C). Receiver operating characteristic (ROC) curves were generated to evaluate the predictive value of the biomarkers, and the area under the curve (AUC) values were used to determine the diagnostic effectiveness in discriminating AF patients from SR patients. The AUC values of the lncRNAs XR_001750763.2, XR_932105.2, NR_038446.1, and ENST00000447009 were 0.798 [95% confidence interval (CI) 0.660–0.935; *p* < 0.01], 0.780 (95% CI 0.629–0.931; *p* < 0.01), 0.725 (95% CI 0.566–0.885; *p* < 0.05), and 0.730 (95% CI 0.572–0.888; *p* < 0.05) for all AF patients, respectively (Figure 7D). These results confirmed that the four lncRNAs were independent predictors of AF. As a key lncRNA in the top 200 ceRNA network, we used the miRanda database to predict the target genes of lncRNA XR_001750763.2. The results showed that XR_001750763.2 had binding interactions with six differentially expressed miRNAs, namely, miR-12136, miR-526b-5p, miR-512-3p, miR-302c-3p, miR-1323, and miR-302b-3p). Among the binding interactions, the most competitive binding sites occurred with miR-302b-3p. Moreover, the miRanda results showed that miR-302b-3p had a binding relationship with the TLR2 inflammatory factor. These findings suggested that lncRNA XR_001750763.2 and TLR2 may act as ceRNAs of miR-302b-3p in AF, and the mechanism of lncRNA XR_001750763.2/miR-302b-3p/TLR2 has important research significance in the occurrence of AF (Figures 7E, F and Supplementary Figure).

**Table 4:** Selected DERNAs in LAA tissues.

LncRNA_id	FC	log2FC	*p*Value	*q*Value	Regulation
XR_001750763.2	25.10239951	4.649753371	0.00016291	0.03332375	Up
XR_932105.2	12.9478958	3.694645755	2.05 × 10^−9^	2.49 × 10^−6^	Up
NR_038446.1	8.119639528	3.02141568	5.54 × 10^−7^	0.00029081	Up
ENST00000447009	9.139720493	3.192150046	0.000106541	0.024118784	Up
miRNA_id					
Has-miR-512-3p	0.428858815	−1.221425321	0.00000915	0.001991071	Down
Has-miR-302c-3p	0.39841765	−1.327646533	0.001069633	0.034465953	Down
Has-miR-302b-3p	0.276407886	−1.855129318	0.000399227	0.024354625	Down
gene_id					
TLR2	2.387902301	1.255743811	0.0000422	0.001975113	Up
JAK3	2.510805036	1.328150008	0.0000272	0.001413451	Up
CCL5	3.193522958	1.675148822	0.000175172	0.005543213	Up
VCAM1	2.270942613	1.18329125	1.75 × 10^−10^	0.000000111	Up

DE, differentially expressed; LAA, left atrial appendage; FC, fold change.

**Table 5:** Comparison of clinical characteristics between the two groups.

Characteristics	SR patients (*n* = 20)	AF patients (*n* = 20)	*p*-value
Age (years)	62.45 ± 8.52	56.85 ± 9.33	0.061
Male, *n* (%)	12 (60.0)	12 (60.0)	1.000
BMI (kg/m^2^)	25.68 ± 3.90	26.43 ± 3.93	0.553
Hypertension, *n* (%)	9 (45.0)	8 (40.0)	0.749
Diabetes mellitus, *n* (%)	4 (20.0)	3 (15.0)	0.677
Stroke, *n* (%)	7 (35.0)	4 (20.0)	0.288
HR (bpm)	77.05 ± 14.30	98.10 ± 22.24	0.001*
SBP (mmHg)	135.90 ± 18.39	128.6 ± 15.15	0.190
DBP (mmHg)	79.20 ± 12.19	81.95 ± 10.18	0.455
Cr (umol/l)	64.65 ± 14.78	64.91 ± 14.10	0.956
ALT(U/l)	19.28 ± 17.70	21.47 ± 18.43	0.711
AST(U/l)	19.55 ± 10.76	17.35 ± 6.64	0.453
TC (mmol/l)	3.73 ± 1.70	3.86 ± 1.22	0.796
TG (mmol/l)	1.93 ± 1.22	1.47 ± 0.78	0.174
HDL-C (mmol/l)	1.29 ± 0.26	1.15 ± 0.18	0.064
LDL-C (mmol/l)	2.76 ± 0.73	2.58 ± 0.65	0.431
LAD (mm)	37.85 ± 5.94	43.65 ± 7.81	0.014*
LVDd (mm)	48.55 ± 8.96	46.20 ± 3.60	0.295
LVEF (%)	58.80 ± 7.51	59.45 ± 4.49	0.748

SR, sinus rhythm; AF, atrial fibrillation; BMI, body mass index; HR, heart rate; SBP, systolic blood pressure; DBP, diastolic blood pressure; Cr, creatinine; ALT, alanine aminotransferase; AST, aspartate transaminase; TC, total cholesterol; TG, total glyceride; HDL-C, high-density lipoprotein cholesterol; LDL-C, low-density lipoprotein cholesterol; RAD, right atrial diameter; LAD, left atrial diameter; LVDd, left ventricular end-diastolic dimension; LVEF, left ventricular ejection fractions. Values are presented as means ± SEM or number (%).

* *p *< 0.05.

## 4. Discussion

AF is a clinically common arrhythmia disease and is associated with increased all-cause mortality, and AF can lead to complications, such as heart failure, thrombosis, and stroke (17, 18). In the present study, the LVEF was decreased in patients with AF, indicating that AF affects the cardiac function of patients, thereby threatening the health of patients. The mechanism of AF is complex. Recently, the pathological function of lncRNAs in AF has been recognized, making them potential candidates for therapeutic targets (19). In the present study, LAA tissues collected from 4 AF and 4 SR patients were used for transcriptome sequencing analysis, and a total of 82 lncRNAs, 18 miRNAs, and 495 mRNAs were significantly DE with |log2FC| > 1 (*p* < 0.05) in AF patients. According to the ceRNA theory, the lncRNA-miRNA-mRNA network of lncRNA XR_001750763.2/miR-302b-3p/TLR2 was constructed to provide further evidence for new biomarkers and mechanisms for AF diagnosis.

LncRNAs, a subclass of ncRNAs, are transcribed from the genome with at least 200 nucleotides (20). Studies found that lncRNAs can function as guides, enhancers, baits, or scaffolds to control gene expression at epigenetic, post-transcriptional, and post-translational levels (21). LncRNAs can participate in the occurrence of cardiovascular disease through interference with neighboring gene expression, regulation of transcription factor activity, and epigenetic modification. For example, lncRNA Safe is enriched in the nuclei of fibroblasts and is elevated in both myocardial infarction and TGF-*β*-induced cardiac fibrosis, and knockdown of lncRNA Safe significantly inhibits the expression of Sfrp2, its nearby protein-coding gene, by regulating the RNA stability in fibroblasts, thus restraining cardiac fibrosis (22). LncRNA NRON expression is significantly downregulated in atrial tissues of AF patients, and studies have found that LncRNA NRON alleviates atrial fibrosis *via* promoting the phosphorylation level of the NFATc3 transcription factor (23). Taken together, these findings suggest that lncRNAs play vital roles in AF and have potential as biomarkers in regulating gene expression.

The expression of lncRNA is highly tissue- and cell type-specific, and the mechanisms of lncRNAs acting as ceRNAs in human AF remain unclear. Chen et al. constructed an AF-related ceRNA network by identifying lncRNAs from the GSE41177 and GSE79768 datasets, and they reported that lncRNA FAM201A acts as a miRNA sponge of miR-33a-3p to upregulate the expression of the Rac family small GTPase 3 (RAC3) target gene, thereby participating in the regulation of AF susceptibility (24). In the present study, we found that the target genes of the lncRNA network were mainly involved in the adaptive immune response, inflammatory response, T/B cell receptor signaling pathway, and calcium signaling pathway. Previous studies have shown that structural remodeling, electrical remodeling, Calcium (Ca^2+^) handling abnormalities, and inflammatory response comprise the basic pathogenesis of AF (25, 26). This also indicates that lncRNAs have the potential to participate in the occurrence of AF. In addition, we also find that lncRNAs have a certain relationship with osteoclast differentiation, Ca^2+^ signals are are known to be crucial for osteoclast differentiation and function (27). Osteoclastic activity is also enhanced with elevated inflammation, artesunate attenuates LPS-induced inflammatory osteoclastogenesis by inhibiting the downstream PLC*γ*1-Ca^2+^-NFATc1 signaling pathway (28). Thus, we speculated whether lncRNA can regulate osteoclast differentiation by regulating Ca^2+^ signaling pathways and inflammatory response, and thus affect the occurrence of AF. This will be discussed further in the future.

We verified the expression levels of several lncRNAs, miRNAs, and mRNAs in the ceRNA network. Few studies have revealed dysregulation of these lncRNAs in the AF population, but previous studies have found an association between these miRNAs and AF. MiRNAs are small noncoding RNAs that negatively regulate target genes and play important roles in the pathophysiology of AF. Wang et al. revealed that miR-302b-3p is downregulated in AF and participates in the pathogenesis of AF by suppressing syndecan 1 (SDC-1) transcript levels, suggesting that miR-302b-3p has a potential diagnostic value to distinguish AF patients from healthy individuals (29). MiR-302c belongs to the miR-302 family, and Bollati et al. implicated miR-302c in the development of cardiac hypertrophy, which can lead to atrial structural remodeling (30). Moreover, miR-302c-3p may target specific sites in the NLR family pyrin domain containing 3 (NLRP3) inflammasome to regulate inflammatory response and endothelial cell pyroptosis (31). Many proinflammatory cytokines, such as interleukin (IL)-6, IL-1β, and tumor necrosis factor (TNF)-*α*, are biomarkers for predicting atrial fibrosis in AF (32). In the ceRNA network of the present study, the CCL5 chemokine was confirmed to be elevated in the serum of AF patients (33). VCAM1 is a protein that is normally involved in the adhesion and transportation process of leukocytes to the interstitium during inflammation. Studies have confirmed that VCAM1 expression is increased in AF patients and can be used as a biomarker to predict the risk of thrombosis in AF (34).

Toll-like receptor 2 (TLR2) is a member of the Toll-like receptor family, and it is used as an important receptor to recognize many exogenous ligands, which causes the release of inflammatory mediators and cytokines, thereby initiating the innate immune response. Previous studies have confirmed that the expression of TLR2 in left atrial blood samples of AF patients is significantly increased and that TLR2 has the potential to be a new biomarker for new-onset AF after acute myocardial infarction (35, 36). In angiotensin II (Ang II)-treated mouse hearts and H9C2 cells, TLR2 deficiency reduces the formation of the TLR2-myeloid differentiation primary response protein 88 (MYD88) complex, which activates the NF-κB pathway, thereby inhibiting the secretion of inflammatory factors, such as IL-1β, IL-6, and TNF-α, ultimately preventing Ang II-induced cardiac remodeling (37).

In the present study, we find that lncRNAs have the potential to become biomarkers of AF. Based on the ceRNA theory, we constructed an lncRNA-miRNA-mRNA network closely related to AF. The lncRNA XR_001750763.2/miR-302b-3p/TLR2 network may be involved in the pathophysiological mechanism of AF by regulating the inflammatory and immune response. Since lncRNA XR_001750763.2 is a newly discovered lncRNA, further experimental exploration and verification are needed in the future.

## 5. Limitations

First, there were only four pairs of samples for transcriptome detection, which may not have provided complete transcriptome information, potentially reducing screening accuracy. Second, future experimental studies are needed to verify the role of the lncRNA XR_001750763.2/miR-302b-3p/TLR2 network in the pathogenesis of AF. Finally, the lncRNA profile between the AF group and SR group was preliminary. There are still many deficiencies in the current understanding of lncRNAs, and the exact mechanism of lncRNAs in the pathogenesis of AF are still unclear. These limitations need to be further explored in the future.

## 6. Conclusion

The present study revealed the specific changes of lncRNAs in the LAA tissues of AF patients compared to SR patients and constructed the XR_001750763.2/miR-302b-3p/TLR2 network based on the ceRNA theory. These findings indicated that lncRNAs have the potential to be diagnostic biomarkers for AF, and the ceRNA network is helpful for future investigations of the regulatory role of lncRNAs in the pathogenesis of AF.

## Data Availability

The datasets presented in this study can be found in online repositories. The names of the repository/repositories and accession number(s) can be found in the article/**Supplementary Material**.
