# Supplementary material for: Integrated analysis of the lncRNA-miRNA-mRNA network based on competing endogenous RNA in atrial fibrillation
Source: Front Cardiovasc Med. 2023 Apr 27;10:1099124. doi: 10.3389/fcvm.2023.1099124 (PMC10174322; doi:10.3389/fcvm.2023.1099124)
Supplement: Supplementary file 1 [file Table7.docx]

Supplementary Material

# Supplementary Tables

**Table S1:** The different expression lncRNA (AF-vs-SR).

**Table S2:** The different expression miRNA (AF-vs-SR).

**Table S3:** The different expression mRNA (AF-vs-SR).

**Table S4**: The co-expression analysis of DEmRNA-DElncRNA.

**Table S5**: The filtering results of ceRNA score and co-expression analysis of DEmRNA-DElncRNA.

**Table S6:** Construction of DElncRNA-DEmiRNA-DEmRNA network.

**Table S7:** GO analysis.

**Table S8:** KEGG analysis

**GEO Accession numbers:** GSE219054.

**Supplementary Figure**

**The miR-302b-3p binding sites on XR_001750763.2(A) and TLR2 (B) were predicted by miRanda.
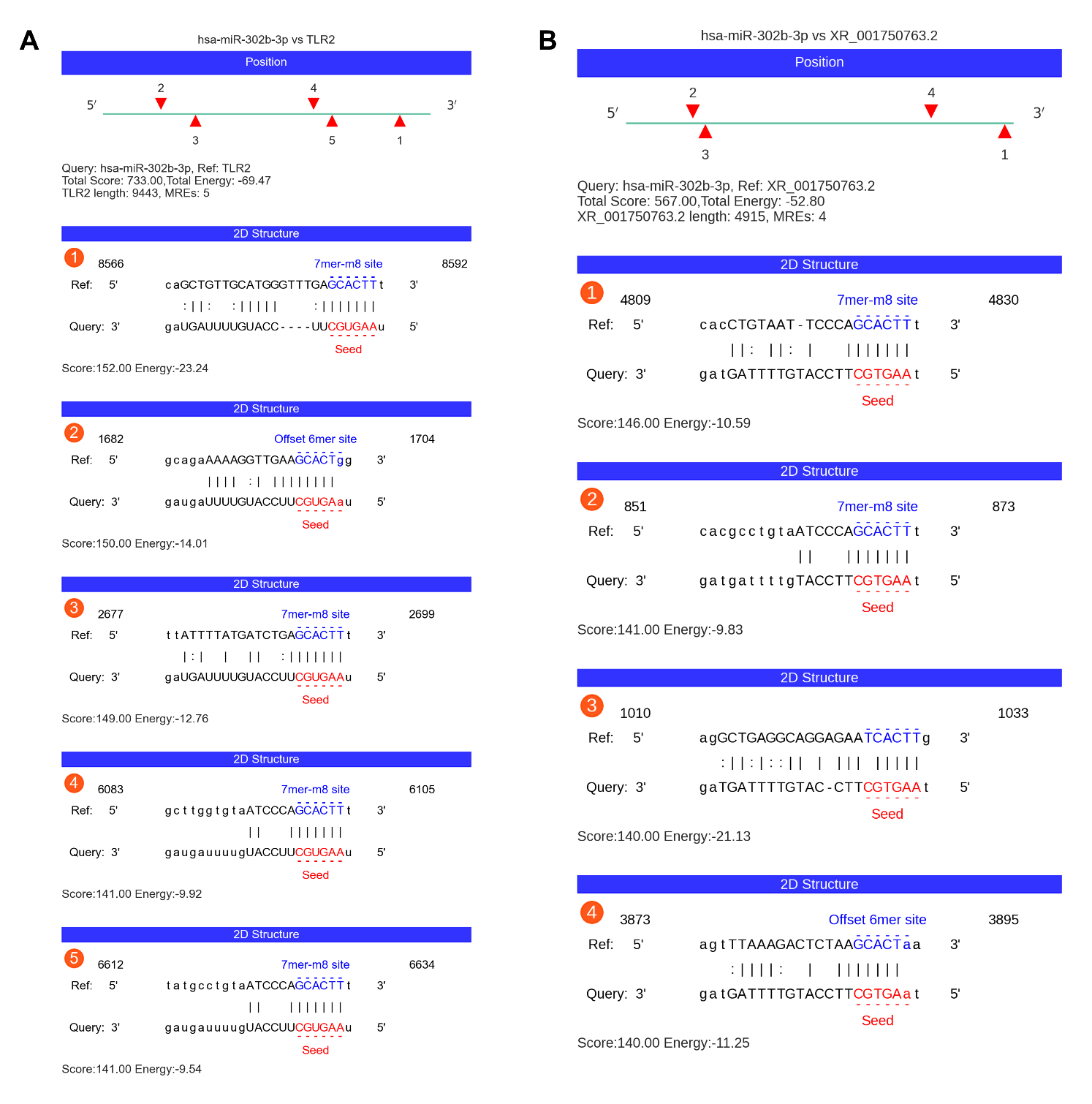
**
